# Effects of Triton X-100 and PEG on the Catalytic Properties and Thermal Stability of Lipase from *Candida Rugosa* Free and Immobilized on Glyoxyl-Agarose

**DOI:** 10.2174/1874091X01711010066

**Published:** 2017-07-31

**Authors:** Rafael F. Perna, Poliana C. Tiosso, Letícia M. Sgobi, Angélica M.S. Vieira, Marcelo F. Vieira, Paulo W. Tardioli, Cleide M.F. Soares, Gisella M Zanin

**Affiliations:** 1 Science and Technology Institute, Federal University of Alfenas, Rod. José Aurélio Vilela, Km 533, 11999, 37715-400 Poços de Caldas, MG, Brazil; 2 Department of Chemical Engineering, State University of Maringá, Av. Colombo, 5790, 87020-900 Maringá, PR.W, Brazil; 3 Department of Food Engineering, State University of Maringá, Av. Colombo, 5790, 87020-900 Maringá, PR.W, Brazil; 4 Departmet of Chemical Engineering, Federal University of São Carlos, Rod. Washington Luis, Km 235, 13565-905, São Carlos, SP, Brazil; 5 Institute of Technology and Research, Tiradentes University, Av. Murilo Dantas, 300, 49032-490 Aracaju, SE, Brazil

**Keywords:** Triton X-100, PEG, Stabilization, *Candida rugosa* Lipase, Immobilization, Glyoxyl-Agarose

## Abstract

**Background:**

*Candida rugosa* Lipase (CRL) shows a very low alkaline stability that comprises its immobilization on glyoxyl-agarose, which requires pH above 10. In this way, an adaptation from the original method was used; an enzyme solution at pH 7 was slowly added at a suspension of glyoxyl-agarose prepared in bicarbonate buffer, pH 10. This change of protocol was enough for allowing the preparation of derivatives actives of CRL on glyoxyl-agarose and verifying the effect of this modified procedure on the properties of the immobilized enzyme. The effect of the additives Triton-X-100 and polyethylene glycol (PEG) on the enzymatic activity recovery and immobilized enzyme stability was evaluated.

**Methods:**

The glyoxyl-agarose support was prepared by etherification of 6% agarose beads with glycidol and further oxidation with sodium periodate. CRL was immobilized covalently on glyoxyl-agarose support in the absence and presence of 1% (w/v) Triton-X-100 or 5 g L^-1^ polyethylene glycol (PEG). The lipolysis activity of the free and immobilized enzyme was determined at 37ºC and pH 7.0, using p-nitrophenyl palmitate (p-NPP) as substrate. Profiles of temperature-activity (37-65ºC, pH 7.0) and pH-activity (6.0-9.5, 37ºC) were evaluated as well as thermal (45ºC and pH 8.0) and operational (15 min batches of p-NPP hydrolysis at 50ºC and pH 8.0) stabilities of free and immobilized CRL.

**Results:**

Using a single modification of the original protocol, the CRL poorly stable under alkaline conditions could be immobilized on glyoxyl-agarose in its active conformation (recovered activity varying from 10.3 to 30.4%). Besides, the presence of a detergent (Triton-X-100) and an enzyme stabilizer (PEG) contributed to the preparation of more active and more stable biocatalysts, respectively. CRL immobilized on glyoxyl-agarose in the presence of PEG was around 5 times more stable than the free CRL and around 3 times more stable than the CRL immobilized on glyoxyl-agarose in absence of PEG. The higher stability of the CRL-glyoxyl derivative prepared in the presence of PEG allowed its reuse in four successive 15 min-batches of p-nitrophenyl palmitate hydrolysis at 50ºC and pH 8.0.

**Conclusion:**

The technique of immobilizing enzymes covalently on glyoxyl-agarose showed promising results for Candida rugosa lipase (CRL). The derivatives prepared in the presence of the additives retained two to three times more activity than those prepared in the absence of additives. The enzyme immobilized in presence of PEG was about three times more stable than the enzyme immobilized in absence of this additive. Maximum catalytic activity of the immobilized CRL (in absence of additives) was observed in a temperature 10ºC above that for the free enzyme and the pH of the maximum activity was maintained in the range 6.5-7.5 for free and immobilized CRL.

## 1 INTRODUCTION

Lipases (E.C. 3.1.1.3) are hydrolytic enzymes that catalyze the hydrolysis of triglycerides to produce mono and diacylglycerides, free fatty acids and glycerol. However, when water activity in the reaction medium is low, the majority of lipases can exert their reverse enzymatic activity, *i.e*., catalysis of esterification, transesterification (*e.g*., acidolysis, alcoholysis, and aminolysis), and interesterification reactions [1-8]. Because of this great catalytic versatility and their high stability in organic and aqueous medium, lipases currently have gained significant share of the world enzyme market.

In general, lipases act at a wide pH range, are stable at high temperatures and do not require cofactors. In addition, they exhibit high specificity and properties of regio-, chemo- and enantioselectivity [9, 10]. These enzymes are the most important among hydrolases due to their multiple applications in industrial processes. Lipases are used in the detergents, medicines, food, textiles, pulp and paper, cosmetics, biodiesel and biosensors industries, as well as applied in the treatment of effluents [10, 11].

Lipases show a typical mechanism of action called interfacial activation [12]. In aqueous media the active site is protected from the reaction medium by an oligopeptide chain known as “lid”, which in the closed form turns the enzyme inactive [13]. The closed form of the lipase (closed lid) may exist in equilibrium with an open form, where the lid has been unlocked, allowing the access of the substrate to the active site [14]. When the enzyme is exposed to a hydrophobic interface (drops of oil, hydrophobic support surfaces, gas bubbles, hydrophobic proteins, lipopolysaccharides, etc), the lid is opened exposing hydrophobic residues located in the internal face of the lid and in the surroundings of the active site, shifting the equilibrium toward the open enzyme structure [12, 13, 15-18]. It have been reported that the presence of detergents in the lipase medium also favors the shifting of the equilibrium towards to the open lid form, allowing the fixation of the lipase on hydrophobic supports in a hyperactivated conformation [19-26]. Besides, lipases may exist in solution as biomolecular aggregates, in which the hydrophobic active centers of the monomers are in close contact, yielding more stable and less active structures. In presence of detergents, these aggregates are dissociated yielding less stable and more active monomers, which may be fixed on glyoxyl supports with the active center turned to the reaction medium [23].

In the last years, studies of lipase immobilization have increased considerably aiming their application in different fields, such as, hydrolysis of oils and fats, and esterification reactions for producing chemical compounds for food, cosmetic and nutraceutical industries [27-29]. Lipases from different microorganisms have been immobilized on various activated supports, such as, octyl-agarose, octadecyl-Sepabeads, DEAE-Sepharose, IDA-Cu^+2^-Sepabeads, MANAE-agarose, CNBr-Sepharose and glyoxyl-agarose [30-40].

Among the great number of activated supports available for immobilizing enzymes [41], active and stable derivatives of many enzymes have been produced using glyoxyl-agarose as support, such as Penicillin G acylase [42, 43], trypsin [44], chymotrypsin [45], thermophilic esterase [46], L-aminoacylase [47], chitosanase [48], carboxypeptiase A [49, 50], alcalase [51], cyclomaltodextrin glucanotransferase [52, 53] and lipase [23, 37, 54, 55].

Glyoxyl-agarose is composed of thick agarose fibers that hold a large number of very stable aldehyde groups linked to the support by very short spacer arms. Under alkaline conditions (pH around 10.0), enzyme immobilization occurs through, at least, a two-point reaction between the protein surface region having higher densities of amino groups and the support aldehyde groups. A more intense multipoint attachment between the immobilized protein and the activated support can be reached, with minimal loss of catalytic activity, by a long-term incubation of the enzyme–support reaction medium under suitable conditions, such as temperature around 20-25ºC. The last step of the immobilization of an enzyme on glyoxyl-agarose is a very mild reduction using sodium borohydride. After that, the enzyme remains attached to the support by means of very stable secondary amino bonds (with physical properties very similar to the initial primary amines) and the non reacted aldehyde groups on the support are transformed into hydrophilic hydroxyl groups [21].

Palomo *et al.* [33] reported that lipase from *Candida rugosa* could not be immobilized on glyoxyl-agarose by the original protocol [45], which requires the suspension of the support in an enzyme solution at pH 10. However, in this work, a simple modification of the original method allowed the immobilization of this lipase in this support: an enzyme solution at pH 7 was slowly added in a suspension of glyoxyl-agarose prepared in bicarbonate buffer 80 mM, pH 10. Besides, CRL was also immobilized on glyoxyl-agarose in the presence of Triton-X-100 (a surfactant that may activate lipases and dissociate bimolecular aggregates) or polyethylene glycol (a stabilizer of enzymes) and their influence on the enzymatic activity recovery and thermal stability of the immobilized enzyme was evaluated.

## 2 MATERIALS AND METHOD

### 2.1 Materials

Lipase from *Candida rugosa* (type VII), glycidol (2,3-epoxy-1-propanol), p-nitrophenyl palmitate (p-NPP) and sodium borohydride were acquired from Sigma Chem. Co (St. Louis, MO, EUA). Sepharose CL-6B was purchased from Amersham Pharmacia Biotech AB (Uppsala, Sweden). Sodium metaperiodate, Triton X-100, gum arabic and polyethylene glycol (PEG- 1450) were purchased from Synth (Diadema, SP, Brazil). All other chemicals were of analytical grade.

### 2.2 Preparation of CRL Glyoxyl-Agarose

#### 2.2.1 Support Activation

The glyoxyl-agarose support with high concentration of aldehyde groups per volume (around 75 µmol mL^-1^) was prepared by etherification of 6% agarose beads with glycidol and further oxidation with sodium periodate [42].

Under gentle stirring and in an ice bath, 105 g of agarose, previously washed with distilled water, were mixed with 30 mL of distilled water, and 50 mL of 1.7 N NaOH, containing 1.425 g sodium borohydride (previously cold prepared). After, 36 mL of glycidol were added dropwise to avoid raising the temperature above 25ºC, and the suspension formed was gently stirred for 12-15 h. The etherified support (glyceryl-agarose) was washed with distilled water, dried by vacuum suction in a sintered glass filter, and resuspended in 895 mL of water (volume ratio support:suspension of 1:10). Sodium metaperiodate (3.21 g) was added and the suspension was gently stirred for 2 h at room temperature. Finally, the glyoxyl-agarose support was washed with water, filtered under vacuum and suck dried.

#### 2.2.2 Immobilization of Enzymes on Glyoxyl-Supports

A mass of 10 g of glyoxyl-agarose were added to 90 mL of 80 mM sodium bicarbonate buffer, pH 10 and maintained under gentle agitation at 25ºC. Next, 10 mL of enzyme solution (0.97 to 4.87 mg of protein/mL) prepared in 50 mM sodium phosphate buffer, pH 7.0, were slowly added. Then, this immobilization suspension was kept under gentle agitation at 25ºC for two or four hours. The enzymatic activity of the initial solution and the final supernatant obtained after the immobilization period was measured to determine the amount of enzyme fixed in the support. After 4 h reaction time, no enzymatic activity was detected in the supernatant and the immobilized lipase derivative was reduced with sodium borohydride (1 g L^-1^, 25ºC, 30 min), as described by Guisán and Blanco [44]. Then, the CRL-derivative was washed with excess distilled water, filtered under vacuum, and suck dried.

CRL was also immobilized in the presence of 1% (w/v) Triton-X-100 [56] or 5 gL^-1^ polyethylene glycol (PEG).

All experiments were performed at least in triplicate and the standard deviations were less than 10%.

### 2.3 Activity Assay

The lipolytic activity of the free and immobilized enzyme was determined at 37ºC, pH 7.0, using p-nitrophenyl palmitate (p-NPP) as substrate [57]. An emulsion of 0.8 mM p-NPP was prepared by mixing 1 mL of p-NPP solution (3 gL^-1^ in isopropanol) and 9 mL of 50 mM phosphate buffer at pH 7.0 containing Triton X-100 and gum arabic (approximately 0.45 and 1.11 gL^-1^, respectively).

For the free CRL, 0.3 mL of an enzyme solution (protein concentration equal to 9.74 µg mL^-1^) was mixed with 2.7 mL of substrate emulsion in a 1 cm optical length cuvette and the absorbance change at 37^o^C was monitored for 10 min at 410 nm in a UV-1203 spectrophotometer (Shimadzu Co., Japan), using pure substrate emulsion as reference. For the immobilized CRL, a mass of approximately 1 mg of immobilized CRL was suspended in 30 mL of substrate emulsion in a batch reactor thermostatized at 37^o^C and samples were withdrawn at regular time intervals up to 10 min reaction for measuring the absorbance change at 410 nm in a UV-1203 spectrophotometer (Shimadzu Co., Japan), using pure substrate emulsion as reference.

The enzymatic activity per mass of enzyme (U g^-1^) was calculated with Equation 1:

(1) $$A=\frac{\Delta \mathrm{ABS}\times V_{R}}{\varepsilon \times m_{E}}\times 10^{3}$$

where ΔABS is the reaction medium absorbance change after 10 min reaction time (cuvette of 1 cm optical length); V_R_ is the total reaction volume (3 mL for the free enzyme and 30 mL for the immobilized enzyme), ε is molar extinction coefficient for the p-NPP (ε = 8.03 x 10^3^ M^-1^ cm^-1^) and m_E_ is the free or immobilized enzyme mass in g. Factor 10^3^ was required for unit conversion (mol of p-nitrophenol to μmol of p-nitrophenol).

One Unit of enzymatic activity (U) was defined as the amount of enzyme that releases 1 μmol of p-nitrophenol per min under the experimental conditions describe above. Under these conditions the free CRL activity was 47.6 ± 3.6 U mg^-1^ of protein. The free enzyme protein content was 32.47 mg g^-1^ as determined by the Bradford’s method [58].

### 2.4 Temperature-activity and pH-activity Profiles

Enzymatic activities of free and immobilized CRL were measured at 37-65ºC (pH 7.0) and at pH 6.5-9.5 (37ºC), using p-NPP as substrate, using the activity method described in the Section 2.3. The molar extinction coefficients (M^-1^ cm^-1^) at different pH were calculated for measurements of activity.

### 2.5 Thermal and Operational Stability

The thermal stability of the CRL-agarose biocatalysts was evaluated at 45ºC and pH 8.0. Biocatalyst suspensions were prepared in 50 mM sodium phosphate buffer, containing Triton X-100 and gum arabic at the concentrations of 0.45 and 1.11 gL^-1^, respectively. Residual activity was measured in 30 min intervals adding 0.8 mM p-NPP solution prepared in isopropanol. After 15 min at 37ºC the absorbance was measured at 410 nm.

The parameters of thermal inactivation were estimated by fitting the model of Sadana and Henley [59] to the experimental data of residual activities *vs*. time (Eq. 2):

(2) $$\frac{A}{A_{0}}=\left(1-\alpha_{1}\right)\exp\left(-k_{1}t\right)+\alpha_{1}$$

where A/A_0_ is the dimensionless residual activity, α_1_ is the ratio of the specific activity of the final state to the initial state, and k_1_ is the first-order deactivation rate constant (time^-1^).

The two-parameter model was fitted to the deactivation data using the Levenberg-Marquardt method of iterative convergence, at 0.95 confidence level. The catalyst half-life (t_½_) was calculated using Eq. 3:

(3) $$t_{\frac{1}{2}}=-\frac{1}{k_{1}}\mathrm{In}\left(\frac{\frac{1}{2}{-\alpha }_{1}}{1-\alpha_{1}}\right)$$

The operational stability of CRL-glyoxyl agarose derivatives prepared in the absence and presence of PEG was also evaluated at 50^o^C, pH 8.0 in reaction medium containing 1 mL of p-NPP (3 gL^-1^ in isopropanol) and 9 mL of 50 mM phosphate buffer, pH 8.0 containing Triton-X-100 (0.45 gL^-1^) and gum Arabic (1.11 gL^-1^). The residual activities of the biocatalysts were assessed in 15 min-intervals using the procedure described in section 2.3.

## 3 RESULTS AND DISCUSSION

### 3.1 Immobilization of CRL on Glyoxyl-Agarose

All CRL-derivatives prepared according to Section 2.2.2 achieved 100% immobilization yield, *i.e.*, all protein offered to the support (1 or 5 mg g^-1^) was immobilized.

Table **1** shows great losses of enzyme activity (65-87%) for CRL immobilized on glyoxyl-agarose in absence or presence of Triton-X-100 or PEG. These losses are probably associated to the low enzyme stability at alkaline pH [33]. In fact, the immobilization of CRL according to the original protocol [42], in absence or presence of additives, resulted in biocatalysts completely inactivated. On the other hand, the slowly enzyme addition was sufficient for allowing the preparation of active CRL derivatives.

The use of Triton-X-100 (1%, w/v) yielded recovered activity higher than that obtained with PEG (5 gL^-1^), which may be associated with the dissociation of bimolecular aggregates (more stable and less active), allowing the immobilization of CRL molecules on glyoxyl-agarose as monomers (less stable and more active) [23]. It have been reported that the addition of a detergent under the critical micellar concentration may increase the lipase activity by shifting the conformational equilibrium of lipases toward the open form. However, detergents may also have negative effects on enzyme activity above a certain concentration [60].

The stabilizing effect of additives on the activity of an enzyme, such as sugars and polyols (*e.g.*, PEG), is based on the reduction of the dielectric constant of the solution compared to the pure water. Consequently, dielectric interactions, as well as hydrophobic ones, between enzyme functional groups should be stronger, making the enzyme molecule more rigid and thus more thermally stable [61]. This effect may have contributed to preserve the CRL activity in alkaline pH, but without allowing the immobilization of the enzyme in the monomeric and more active form because of the greater rigidity of the molecule in the presence of PEG impairing the bimolecular aggregate dissociation. This may explain the lower activity recovered when PEG was used as additive instead Triton-X-100 for CRL immobilization.

### 3.2 Profiles of Activity *vs* pH and Temperature

The activity-pH profile for free and immobilized CRL was evaluated in the range 6.0-10.5. Fig. (**1**) shows that the pH of maximum activity was 7.0 for free CRL and 6.5-7.5 for immobilized CRL in the presence of Triton-X-100 (1%, w/v), which is consistent with the fact that glyoxyl-agarose is a non-charged support, therefore not changing the microenvironment closed to the immobilized lipase [62]. Similar behavior was also reported by Pereira *et al.* [63] for CRL immobilized on chitosan and by Soares *et al.* [64] for CRL encapsulated into hydrophobic sol-gel matrix.

The catalytic activity for free and immobilized CRL was measured at pH 7.0 in the temperature range of 37-60ºC. Fig. (**2**) shows that for immobilized lipases the temperature of maximum catalytic activity occurs between 45 and 50ºC (except for CRL immobilized in presence of PEG), while for free CRL this maximum is found approximately at 40ºC. Similar behavior has been previously reported [63-66]. The 5-10ºC raise in the maximum activity temperature for immobilized CRL demonstrates its greater stability as consequence of the 3D-structure rigidification promoted probably by the formation of multiple covalent links enzyme-support.

### 3.3 Thermal and Operational Stability

Fig. (**3**) shows the inactivation profiles of free and immobilized CRL at 45ºC and pH 8.0 during a time period of 120 min. CRL free and immobilized on glyoxyl-agarose (presence of Triton-X-100) showed similar behavior of inactivation, rapidly dropping its initial activity to around 30% after 30 min incubation. On the other hand, in same time interval, CRL immobilized in the presence of PEG retained around 95% of its initial activity.

The half-lives of CRL immobilized on glyoxyl-agarose in the absence and presence of PEG were estimated as 34.5 and 109 min, respectively, representing a stabilization factor around 3 times. Compared to the free CRL (half-life around 18.5 min), the CRL immobilized on glyoxyl-agarose in presence of PEG showed to be around 6 times more stable.

Probably, CRL was immobilized on glyoxyl-agarose (absence and presence of PEG) as bimolecular aggregates (more stable and less active) and CRL was immobilized on glyoxyl-agarose (presence of Triton-X-100) as monomers (more active and less stable).

The operational stability is a parameter of fundamental importance when it is desired to use the immobilized enzyme industrially [67]. Therefore, the stability of the immobilized biocatalysts prepared in the absence and presence of PEG (the most thermally stable biocatalysts) were evaluated in the reaction medium of p-NPP hydrolysis at 50ºC and pH 8.0. Fig. (**4**) shows that CRL immobilized on glyoxyl-agarose in the presence of PEG was around 3 times more stable than the one immobilized in absence of PEG. This result shows the stabilizing effect of PEG, allowing the immobilization of CRL on glyoxyl-agarose in a rigid and stable conformation.

## CONCLUSION

The technique of immobilizing enzymes covalently on glyoxyl-agarose showed promising results for *Candida rugosa* lipase (CRL). Using a single modification of the original protocol, *i.e.*, adding slowly the enzyme solution prepared at pH 7.0 in the support suspension prepared at pH 10.0 instead of preparing the enzyme solution directly at pH 10.0, the CRL poorly stable under alkaline conditions could be immobilized on glyoxyl-agarose in its active conformation (recovered activity varying from 10.3 to 30.4%). Besides, the presence of a detergent (Triton-X-100) and an enzyme stabilizer (PEG) contributed to the preparation of more active and more stable biocatalysts, respectively. CRL immobilized on glyoxyl-agarose in the presence of PEG was around 5 times more stable than the free CRL and around 3 times more stable than the CRL immobilized on glyoxyl-agarose in absence of PEG. The higher stability of the CRL-glyoxyl derivative prepared in the presence of PEG allowed its reuse in four successive 15 min-batches of p-nitrophenyl palmitate hydrolysis at 50ºC and pH 8.0.

## Figures and Tables

**Fig. (1):**
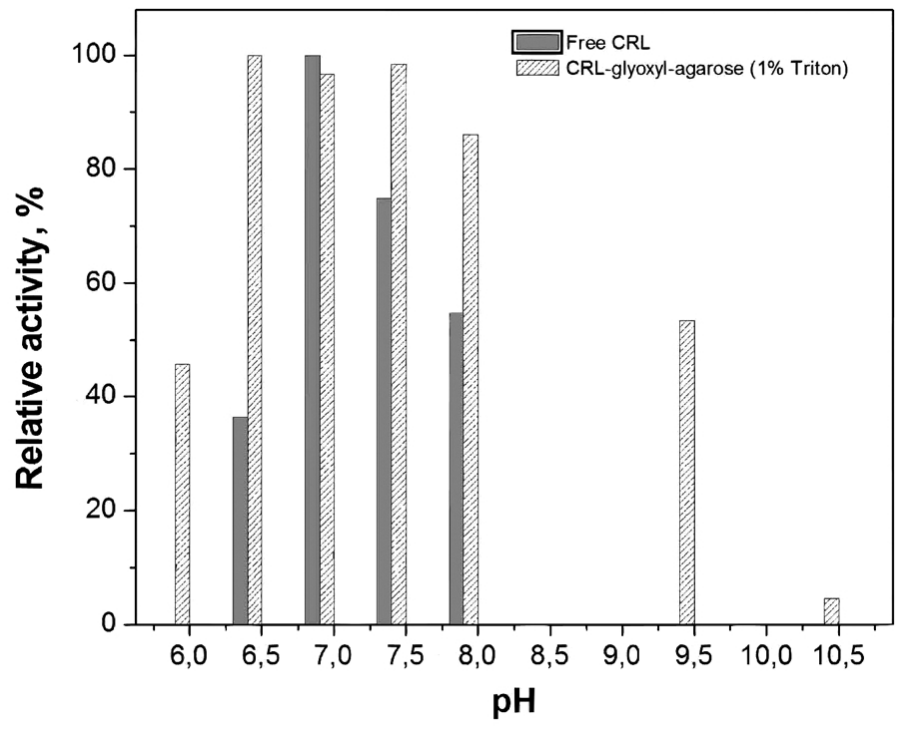
Activity-pH profiles of CRL free and immobilized on glyoxyl-agarose in absence and presence of Triton-X-100 and PEG. Activities were measured at 37ºC and the maximum activity was taken as 100%.

**Fig. (2):**
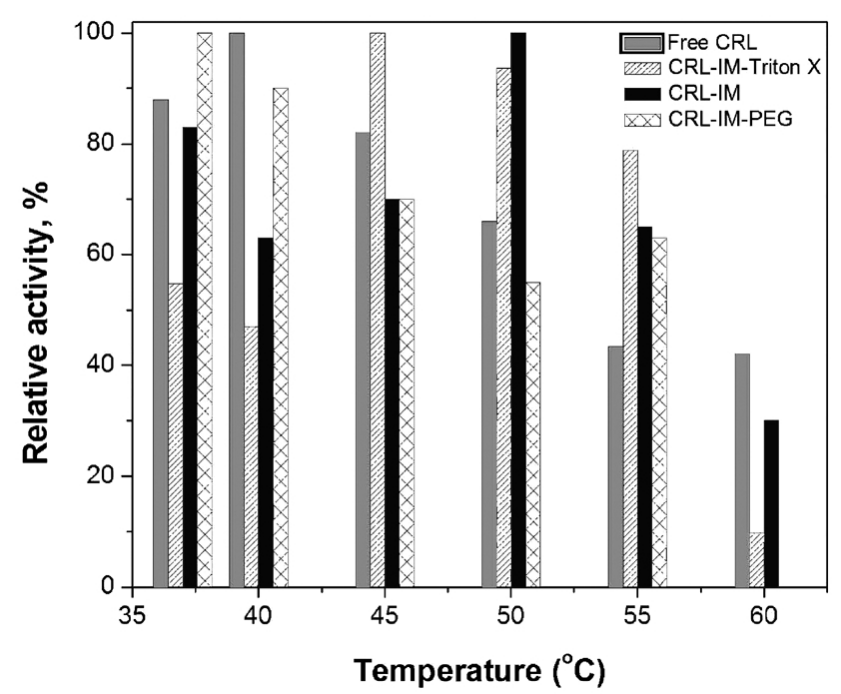
Activity-temperature profiles of CRL free and immobilized on glyoxyl-agarose in absence and presence of Triton-X-100 and PEG. Activities were measured at pH 7.0 and the maximum activity was taken as 100%.

**Fig. (3):**
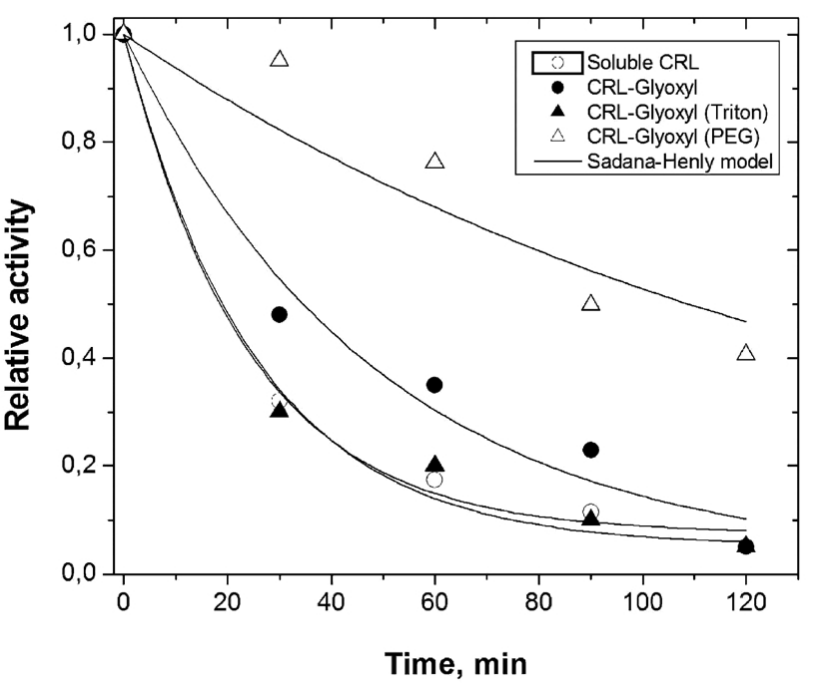
Thermal stability at 45 ºC and pH 8.0 for CRL free and immobilized on glyoxyl-agarose (absence and presence of Triton-X-100 and PEG). Half-lives estimated by Sadana-Henley model [57]: free CRL (t_1/2_ = 18.5 min); CRL-glyoxyl (t_1/2_ = 34.5 min); CRL-glyoxyl, presence of Triton-X-100 (t_1/2_ = 19.0 min); CRL-glyoxyl, presence of PEG (t_1/2_ = 109 min).

**Fig. (4):**
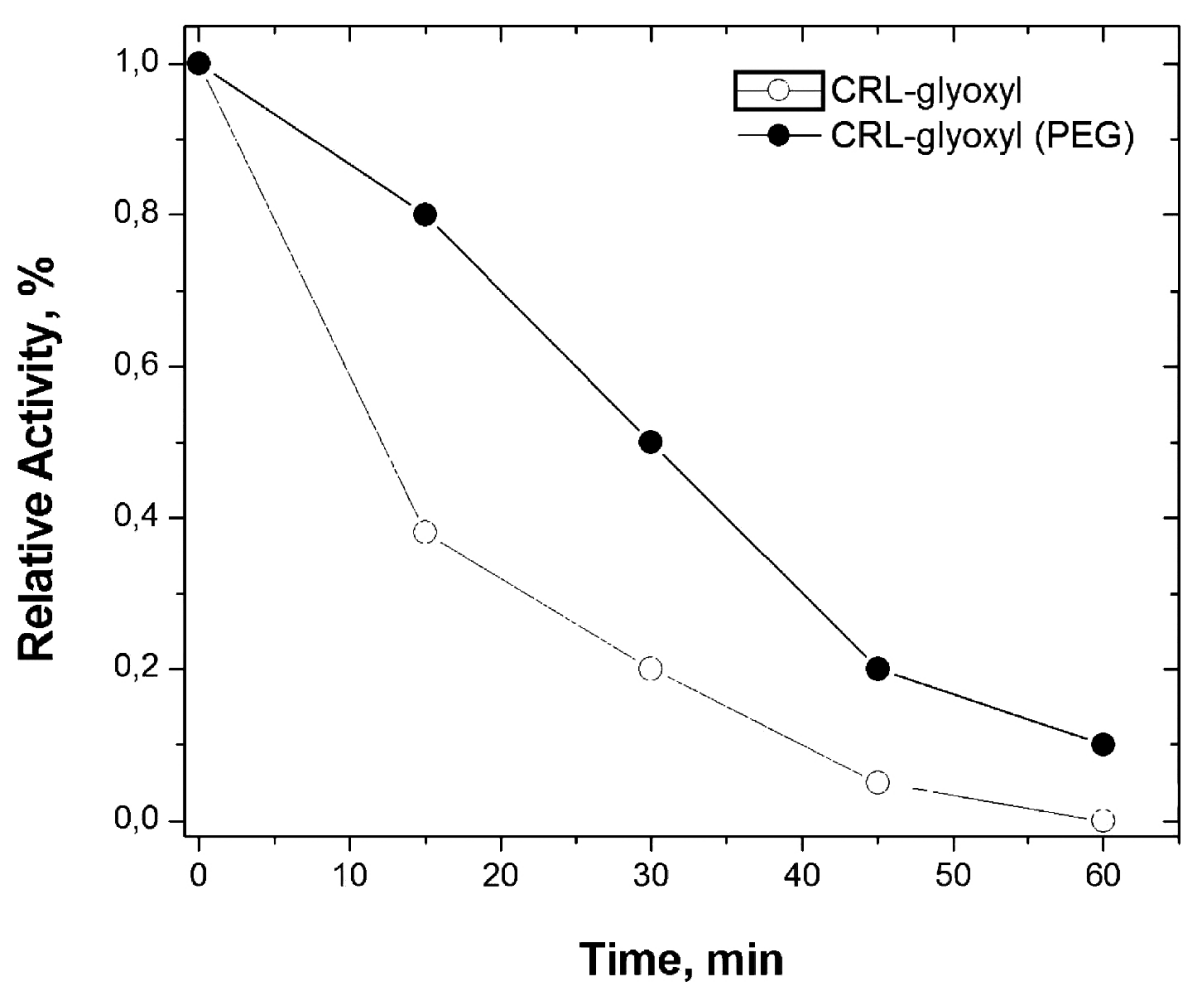
Thermal stability at 50ºC and pH 8.0 for CRL immobilized on glyoxyl-agarose (absence and presence of PEG) in the reaction medium of p-NPP hydrolysis. Half-lives estimated by Sadana-Henley model [59]: absence of PEG (t_1/2_ = 11 min); presence of PEG (t_1/2_ = 30 min).

**Table 1:** CRL immobilization on glyoxyl-agarose (25^o^C, pH 10).

**Additive**	**Enzyme Activity Offered per Mass of Support** **(U g^-1^)**	**IE^b^ Activity per Mass of Support** **(U g^-1^)**	**Activity Recovery^d^** **(%)**
Absence^a^	46.2	5.8	12.6
Triton-X-100 (1%, w/v)^c^	231.8	82.1	35.4
PEG (g L^-1^)^c^	231.8	58.2	25.1

^a^ Enzyme load offered as protein mass per support mass, about 1 mg g^-1^ and reaction time of 2h.

^b^ IE – Immobilized enzyme (derivative CRL-agarose).

^c^ Enzyme load offered as protein per support mass, about 5 mg g^-1^ and reaction time of 4h.

^d^ Activity recovery was defined as the ratio of the immobilized enzyme activity and the enzyme activity offered for immobilization per gram of support. Results are expressed as average of triplicate assays (standard deviations less than 10%). Activities were measured at 37^o^C and pH 7.0 using olive oil as substrate (Section 2.3 – Activity assay).

## References

[1] Castro H.F., Anderson W.A. (1995). Fine chemicals by biotransformation using lipases.. Quim. Nova.

[2] Costa V.E., Amorim H.L. (1999). The use of lipases as agents of kinetic resolution of enantiomers in organic synthesis: general aspects of solvent’s influence.. Quim. Nova.

[3] Bora L., Gohain D., Das R. (2013). Recent advances in production and biotechnological applications of thermostable and alkaline bacterial lipases.. J. Chem. Technol. Biotechnol..

[4] Gupta R., Kumari A., Syal P., Singh Y. (2015). Molecular and functional diversity of yeast and fungal lipases: their role in biotechnology and cellular physiology.. Prog. Lipid Res..

[5] Chen H., Wu J., Yang L., Xu G. (2014). Characterization and structure basis of *Pseudomonas alcaligenes* lipase’s enantiopreference towards d,l-menthyl propionate.. J. Mol. Catal., B Enzym..

[6] Villalba M., Verdasco-Martín C.M., Dos Santos J.C., Fernandez-Lafuente R., Otero C. (2016). Operational stabilities of different chemical derivatives of Novozym 435 in an alcoholysis reaction.. Enzyme Microb. Technol..

[7] Mouad A.M., Taupin D., Lehr L., Yvergnaux F., Porto A.L. (2016). Aminolysis of linoleic and salicylic acid derivatives with *Candida antarctica* lipase B: A solvent-free process to obtain amphiphilic amides for cosmetic application.. J. Mol. Catal., B Enzym..

[8] Verdasco-Martín C., Villalba M., Dos Santos J.C., Tobajas M., Fernandez-Lafuente R., Otero C. (2016). Effect of chemical modification of Novozym 435 on its performance in the alcoholysis of camelina oil.. Biochem. Eng. J..

[9] Villeneuve P., Muderhwa J.M., Graille J., Haas M.J. (2000). Customizing lipases for biocatalysis: a survey of chemical, physical and molecular biological approaches.. J. Mol. Catal., B Enzym..

[10] Hasan F., Shah A.A., Hameed A. (2006). Industrial applications of microbial lipases.. Enzyme Microb. Technol..

[11] Houde A., Kademi A., Leblanc D. (2004). Lipases and their industrial applications: an overview.. Appl. Biochem. Biotechnol..

[12] Verger R. (1997). Interfacial activation’ of lipases: facts and artifacts.. Trends Biotechnol..

[13] Brzozowski A.M., Derewenda U., Derewenda Z.S., Dodson G.G., Lawson D.M., Turkenburg J.P., Björkling F., Huge-Jensen B., Patkar S.A., Thim L. (1991). A model for interfacial activation in lipases from the structure of a fungal lipase-inhibitor complex.. Nature.

[14] Kim K.K., Song H.K., Shin D.H., Hwang K.Y., Suh S.W. (1997). The crystal structure of a triacylglycerol lipase from *Pseudomonas cepacia* reveals a highly open conformation in the absence of a bound inhibitor.. Structure.

[15] Otero C., Fernández-Péreza M., Hermosob J.A., Ripollb M.M. (2005). Activation in the family of *Candida rugosa* isolipases by polyethylene glycol.. J. Mol. Catal., B Enzym..

[16] Nagarajan S. (2012). New tools for exploring “old friends-microbial lipases”.. Appl. Biochem. Biotechnol..

[17] Ramos E.Z., Miotti Júnior R.H., Castro P.F., Tardioli P.W., Mendes A.A., Fernandéz-Lafuente R., Hirata D.B. (2015). Production and immobilization of *Geotrichum candidum* lipase via physical adsorption on eco-friendly support: characterization of the catalytic properties in hydrolysis and esterification reactions.. J. Mol. Catal., B Enzym..

[18] Manoel E.A., Dos Santos J.C., Freire D.M., Rueda N., Fernandez-Lafuente R. (2015). Immobilization of lipases on hydrophobic supports involves the open form of the enzyme.. Enzyme Microb. Technol..

[19] Fernandez-Lorente G., Palomo J.M., Cabrera Z., Fernandez-Lafuente R., Guisán J.M. (2007). Improved catalytic properties of immobilized lipases by the presence of very low concentrations of detergents in the reaction medium.. Biotechnol. Bioeng..

[20] Helistö P., Korpela T. (1998). Effects of detergents on activity of microbial lipases as measured by the nitrophenyl alkanoate esters method.. Enzyme Microb. Technol..

[21] Mateo C., Palomo J.M., Fuentes M., Betancor L., Grazu V., López-Gallego F., Pessela B.C., Hidalgo A., Fernández-Lorente G., Fernández-Lafuente R., Guisán J.M. (2006). Glyoxyl agarose: A fully inert and hydrophilic support for immobilization and high stabilization of proteins.. Enzyme Microb. Technol..

[22] Mogensen J.E., Sehgal P., Otzen D.E. (2005). Activation, inhibition, and destabilization of *Thermomyces lanuginosus* lipase by detergents.. Biochemistry.

[23] Lima L.N., Aragon C.C., Mateo C., Polomo J.M., Giordano R.L., Tardioli P.W., Guisán J.M., Fernandez-Lorente G. (2013). Immobilization and stabilization of a bimolecular aggregate of the lipase from *Pseudomonas fluorescens* by multipoint covalent attachment.. Process Biochem..

[24] Garcia-Galan C., Dos Santos J.C., Barbosa O., Torres R., Pereira E.B., Corberan V.C., Gonçalves L.R., Fernandez-Lafuente R. (2014). Tuning of Lecitase features via solid-phase chemical modification: Effect of the immobilization protocol.. Process Biochem..

[25] Dos Santos J.C., Garcia-Galan C., Rodrigues R.C., De Sant’ Ana H.B., Gonçalves L.R., Fernandez-Lafuente R. (2014). Improving the catalytic properties of immobilized Lecitase via physical coating with ionic polymers.. Enzyme Microb. Technol..

[26] Dos Santos J.C., Garcia-Galan C., Rodrigues R.C., De Sant’Ana H.B., Gonçalves L.R., Fernandez-Lafuente R. (2014). Stabilizing hyperactivated lecitase structures through physical treatment with ionic polymers.. Process Biochem..

[27] Lage F.A., Bassi J.J., Corradini M.C., Todero L.M., Luiz J.H., Mendes A.A. (2016). Preparation of a biocatalyst via physical adsorption of lipase from Thermomyces lanuginosus on hydrophobic support to catalyze biolubricant synthesis by esterification reaction in a solvent-free system.. Enzyme Microb. Technol..

[28] Souza T.C., Fonseca T.S., Costa J.A., Rocha M.V., Mattos M.C., Fernandez-Lafuente R., Gonçalves L.R., Dos Santos J.C. (2016). Cashew apple bagasse as a support for the immobilization of lipase B from *Candida antarctica*: Application to the chemoenzymatic production of (R)-Indanol.. J. Mol. Catal., B Enzym..

[29] Costa V.M., Souza M.C., Fechine P.B., Macedo A.C., Gonçalves L.R. (2016). Nanobiocatalytic systems based on lipase-Fe_3_O_4_ and conventional systems for isoniazid synthesis: A comparative study.. Braz. J. Chem. Eng..

[30] Cunha A.G., Fernández-Lorente G., Bevilaqua J.V., Destain J., Paiva L.M., Freire D.M., Fernández-Lafuente R., Guisán J.M. (2008). Immobilization of *Yarrowia lipolytica* lipase-a comparison of stability of physical adsorption and covalent attachment techniques.. Appl. Biochem. Biotechnol..

[31] Fernández-Lorente G., Ortiz C., Segura R.L., Fernández-Lafuente R., Guisán J.M., Palomo J.M. (2005). Purification of different lipases from *Aspergillus niger* by using a highly selective adsorption on hydrophobic supports.. Biotechnol. Bioeng..

[32] Nieto I., Rocchietti S., Ubiali D., Speranza G., Morelli C.F., Fuentes 
i.e.
, Alcântara A.R., Terreni M. (2005). Immobilization of different protein fractions from *Rhizomucor miehei* lipase crude extract–Enzymatic resolution of (R,S)-2-tetralol.. Enzyme Microb. Technol..

[33] Palomo J.M., Munoz G., Fernandez-Lorente G., Mateo C., Fernandez-Lafuente R., Guisán J.M. (2002). Interfacial adsorption of lipases on very hydrophobic support (octadecyl-Sepabeads): immobilization, hyperactivation and stabilization of the open form of lipases.. J. Mol. Catal., B Enzym..

[34] Palomo J.M., Mateo C., Fernández-Lorente G., Solares L.F., Diaz M., Sanchez V.M., Bayod M., Gotor V., Guisán J.M., Fernandez-Lafuente R. (2003). Resolution of (±)-5-substituted-6-(5-chloropyridin-2-yl)-7-oxo 5, dihydropyrrolo[3,4b]pyrazine derivatives-precursors of (S)-(+)-Zopiclone, catalyzed by immobilized *Candida antarctica* B lipase in aqueous media.. Tetrahedron Asymmetry.

[35] Palomo J.M., Segura R.L., Fernández-Lorente G., Pernas M., Rua M.L., Guisán J.M., Fernández-Lafuente R. (2004). Purification, immobilization, and stabilization of a lipase from *Bacillus thermocatenulatus* by interfacial adsorption on hydrophobic supports.. Biotechnol. Prog..

[36] Wilson L., Palomo J.M., Fernandez-Lorente G., Illanes A., Guisán J.M., Fernandez-Lafuente R. (2006). Improvement of the functional properties of a thermostable lipase from *Alcaligenes* sp. via strong adsorption on hydrophobic supports.. Enzyme Microb. Technol..

[37] Rodrigues D.S., Mendes A.A., Adriano W.S., Gonçalves L.R., Giordano R.L. (2008). Multipoint covalent immobilization of microbial lipase on chitosan and agarose activated by different methods.. J. Mol. Catal., B Enzym..

[38] Ali Z., Tian L., Zhao P., Zhang B., Ali N., Khan M., Zhang Q. (2016). Immobilization of lipase on mesoporous silica nanoparticles with hierarchical fibrous pore.. J. Mol. Catal., B Enzym..

[39] Rueda N., Dos Santos J.C., Ortiz C., Barbosa O., Fernandez-Lafuente R., Torres R. (2016). Chemical amination of lipases improves their immobilization on octyl-glyoxyl agarose beads.. Catal. Today.

[40] Cai C., Gao Y., Liu Y., Zhong N., Liu N. (2016). Immobilization of Candida antarctica lipase B onto SBA-15 and their application in glycerolysis for diacylglycerols synthesis.. Food Chem..

[41] Dos Santos J.C., Barbosa O., Ortiz C., Berenguer-Murcia A., Rodrigues R.C., Fernandez-Lafuente R. (2015). Importance of the support properties for immobilization or purification of enzymes.. ChemCatChem.

[42] Guisán J.M. (1988). Aldehyde-agarose gels as activated supports for immobilization-stabilization of enzymes.. Enzyme Microb. Technol..

[43] Pereira G.H., Guisán J.M., Giordano R.L. (1997). Multi-point immobilization of penicillin G acylase on silica-glyoxyl: Influence of the degree of activation.. Braz. J. Chem. Eng..

[44] Guisán J.M., Blanco R.M. (1987). Stabilization of trypsin by multiple-point attachment to aldehyde-agarose gels.. Ann. N. Y. Acad. Sci..

[45] Guisán J.M., Bastida A., Cuesta C., Fernandez-Lufuente R., Rosell C.M. (1991). Immobilization-stabilization of α-chymotrypsin by covalent attachment to aldehyde-agarose gels.. Biotechnol. Bioeng..

[46] Fernandez-Lafuente R., Cowan D.A., Wood A.N. (1995). Hyperstabilization of a thermophilic esterase by multipoint covalent attachment.. Enzyme Microb. Technol..

[47] Toogood H.S., Taylor J.N., Brown R.C., Taylor S.J., Maccague R., Littlechild A. (2002). Immobilization of the thermostable l-aminoacylase from *thermococcus litoralis* to generate a reusable industrial biocatalyst.. Biocatal. Biotransform..

[48] Ichikawa S., Tanako K., Kuroiwa Y., Hiruta T., Sato S., Mukataka S. (2002). Imobilization of chitosanase by multipoint covalent attachment to agar gel support.. J. Biosci. Bioeng..

[49] Pedroche J., Mar Yust M., Giróncalle J., Vioque J., Alaiz M., Mateo C., Guisán J.M., Lillán F. (2002). Stabilization-immobilization of carboxypeptidase A to aldehyde-agarose gels. A pratical example in the hydrolysis of casein.. Enzyme Microb. Technol..

[50] Tardioli P.W., Fernández-Lafuente R., Guisán J.M., Giordano R.L. (2003). Design of new immobilized-stabilized carboxypeptidase a derivative for production of aromatic free hydrolysates of proteins.. Biotechnol. Prog..

[51] Tardioli P.W., Pedroche J., Giordano R.L., Fernández-Lafuente R., Guisán J.M. (2003). Hydrolysis of proteins by immobilized-stabilized alcalase-glyoxyl agarose.. Biotechnol. Prog..

[52] Tardioli P.W., Zanin G.M., Moraes F.F. (2006). Characterization of *Thermoanaerobacter* cyclomaltodextrin glucanotransferase immobilized on glyoxyl-agarose.. Enzyme Microb. Technol..

[53] Ferrarotti S.A., Bolivar J.M., Mateo C., Wilson L., Guisan J.M., Fernandez-Lafuente R. (2006). Immobilization and stabilization of a cyclodextrin glycosyltransferase by covalent attachment on highly activated glyoxyl-agarose supports.. Biotechnol. Prog..

[54] Osuna Y., Sandoval J., Saade H., López R.G., Martinez J.L., Colunga E.M., de la Cruz G., Segura E.P., Arévalo F.J., Zon M.A., Fernández H., Ilyina A. (2015). Immobilization of *Aspergillus niger* lipase on chitosan-coated magnetic nanoparticles using two covalent-binding methods.. Bioprocess Biosyst. Eng..

[55] dos Santos J.C., Rueda N., Gonçalves L.R., Fernandez-Lafuente R. (2015). Tuning the catalytic properties of lipases immobilized on divinylsulfone activated agarose by altering its nanoenvironment.. Enzyme Microb. Technol..

[56] Palomo J.M., Ortiz C., Fuentes M., Fernandez-Lorente G., Guisan J.M., Fernandez-Lafuente R. (2004). Use of immobilized lipases for lipase purification via specific lipase-lipase interactions.. J. Chromatogr. A.

[57] Kordel M., Hofmann B., Schomburg D., Schmid R.D. (1991). Extracellular lipase of *Pseudomonas sp.* strain ATCC 21808: purification, characterization, crystallization, and preliminary X-ray diffraction data.. J. Bacteriol..

[58] Bradford M.M. (1976). A rapid and sensitive method for the quantitation of microgram quantities of protein utilizing the principle of protein-dye binding.. Anal. Biochem..

[59] Sadana A., Henley J.P. (1987). Single-step unimolecular non-first-order enzyme deactivation kinetics.. Biotechnol. Bioeng..

[60] Fernandez-Lorente G., Palomo J.M., Cabrera Z., Fernandez-Lafuente R., Guisán J.M. (2007). Improved catalytic properties of immobilized lipases by the presence of very low concentrations of detergents in the reaction medium.. Biotechnol. Bioeng..

[61] Gupta M.N. (1991). Thermostabilization of proteins.. Biotechnol. Appl. Biochem..

[62] Blanch H.W., Clarck D.S. (1997). Biochemical Engineering..

[63] Pereira E.B., De Castro H.F., De Moraes F.F., Zanin G.M. (2001). Kinetic studies of lipase from *Candida rugosa*: a comparative study between free and immobilized enzyme onto porous chitosan beads.. Appl. Biochem. Biotechnol..

[64] Soares C.M., Santos O.A., Castro H.F., Moraes F.F., Zanin G.M. (2004). Studies on lipase immobilization in hydrophobic sol-gel matrix.. Appl. Biochem. Biotechnol..

[65] Bagi K., Simon M., Szajá B. (1997). Immobilization and characterization of porcine pancreas lipase.. Enzyme Microb. Technol..

[66] Pereira E.B., Zanin G.M., Castro H.F. (2003). Immobilization and catalytic properties of lipase on chitosan for hydrolysis and esterification reactions.. Braz. J. Chem. Eng..

[67] Kennedy J.F., White C.A., Wiseman A. (1985). Handbook of Enzyme Biotechnology..

